# Clonal Heterogeneity in the Neuronal and Glial Differentiation of Dental Pulp Stem/Progenitor Cells

**DOI:** 10.1155/2016/1290561

**Published:** 2016-05-26

**Authors:** Fraser I. Young, Vsevolod Telezhkin, Sarah J. Youde, Martin S. Langley, Maria Stack, Paul J. Kemp, Rachel J. Waddington, Alastair J. Sloan, Bing Song

**Affiliations:** ^1^Oral and Biomedical Sciences, School of Dentistry, Cardiff University, Heath Park, Cardiff CF14 4XY, UK; ^2^Neuroscience and Mental Health Research Institute, School of Medicine, Cardiff University, Hadyn Ellis Building, Maindy Road, Cardiff CF24 4HQ, UK; ^3^Cardiff School of Biosciences, Cardiff University, Sir Martin Evans Building, Museum Avenue, Cardiff CF10 3AX, UK; ^4^Department of Neuroscience, Physiology & Pharmacology, University College London, London WC1E 6BT, UK

## Abstract

Cellular heterogeneity presents an important challenge to the development of cell-based therapies where there is a fundamental requirement for predictable and reproducible outcomes. Transplanted Dental Pulp Stem/Progenitor Cells (DPSCs) have demonstrated early promise in experimental models of spinal cord injury and stroke, despite limited evidence of neuronal and glial-like differentiation after transplantation. Here, we report, for the first time, on the ability of single cell-derived clonal cultures of murine DPSCs to differentiate* in vitro* into immature neuronal-like and oligodendrocyte-like cells. Importantly, only DPSC clones with high nestin mRNA expression levels were found to successfully differentiate into Map2 and NF-positive neuronal-like cells. Neuronally differentiated DPSCs possessed a membrane capacitance comparable with primary cultured striatal neurons and small inward voltage-activated K^+^ but not outward Na^+^ currents were recorded suggesting a functionally immature phenotype. Similarly, only high nestin-expressing clones demonstrated the ability to adopt Olig1, Olig2, and MBP-positive immature oligodendrocyte-like phenotype. Together, these results demonstrate that appropriate markers may be used to provide an early indication of the suitability of a cell population for purposes where differentiation into a specific lineage may be beneficial and highlight that further understanding of heterogeneity within mixed cellular populations is required.

## 1. Introduction

Stem cell heterogeneity poses a significant obstacle to the clinical implementation of cell-based therapies. Mixed cultures may contain a combination of stem cells with a broad range of differentiation potentials and long term proliferative abilities, as well as more lineage-committed progenitor cells. Such variability amongst cells in the same transplantable population can lead to the adoption of adverse phenotypes, potentially limiting improvements in outcome.

Dental Pulp Stem/Progenitor Cells (DPSCs) possess typical mesenchymal progenitor properties* in vitro*, demonstrating the ability to differentiate into odontoblasts/osteoblasts, adipocytes, and chondrocytes [1–3]. Furthermore, endothelial, myogenic, hepatocytic, and melanocytic differentiation capabilities have also been reported, suggesting a diverse range of potential therapeutic applications [4–8]. However, DPSCs represent a highly heterogeneous population of cells with distinct clonal differences in proliferation and mineralisation capabilities [1, 9]. Such variability could potentially hinder progress in the development of DPSC-based treatments. Nevertheless, transplanted mixed populations of DPSCs have demonstrated promise at improving functional outcome in experimental models of spinal cord injury, stroke, and Parkinson's disease [10–15]. These effects are mostly protective and mediated through the release of supportive growth factors. It is, as yet, unclear whether DPSCs can differentiate into, and functionally compensate for, neuronal and glial cell types after transplantation.* In vitro* studies have indicated potential for rodent and human DPSCs to differentiate into neuronal-like cells [16]. Furthermore, independent studies have described a degree of functionality of such neuronally differentiated DPSCs [17–21]. However, only a small fraction of cells within such cultures develop a functional phenotype [18]. Similarly, outcomes may differ between patient samples subjected to the same differentiation protocol [22]. Understanding the cellular biology behind such heterogeneity poses a substantial challenge to researchers. The identification of an appropriate marker of neural differentiation capabilities, for which to screen DPSC cultures beforehand, could help to minimise such variability between studies resulting in more defined and predictable outcomes.

In this study, single cell-derived clonal populations of murine DPSCs (mDPSCs) were isolated and differences in the expression of early stage neural markers identified. Only clones with high levels of nestin expression were found to differentiate into immature neuronal-like cells, displaying minimal electrical activity. Similarly, a novel differentiation protocol was developed for the derivation of oligodendrocyte-like cells from high nestin-expressing mDPSC clones. Together, these findings suggest that nestin may act as a suitable marker for use in assessing the ability of mDPSCs to differentiate into neuronal-like and glial-like cells* in vitro*.

## 2. Experimental Procedures

### 2.1. Isolation of mDPSCs

All procedures were approved by the Cardiff University Biological Standards Office. For each DPSC isolation, the incisor pulpal tissue of 4-5 × 21–28-day-old C57/Bl6 mice, sacrificed by CO_2_ asphyxiation in accordance with Schedule 1 of the UK Animals (Scientific Procedures) Act 1986, was pooled. Following collagenase/dispase digestion to a cellular suspension, the preferential adherence to fibronectin selection technique was used to select for progenitor cells by isolating cells of more immature phenotypes based on *β*1 integrin functionality [23, 24]. After 12 days of primary culture, individual colonies of fibronectin-adherent cells, displaying typical DPSC bipolar fibroblastic-like morphology and numbering greater than 32 cells, were selected for clonal isolation and expansion as described previously using cloning rings [25]. Clonal DPSCs were subsequently expanded in *α*MEM supplemented with 100 units/mL penicillin, 100 *μ*g/mL streptomycin, and 20% (v/v) heat-inactivated foetal bovine serum (all from Life Technologies) and 100 *μ*M l-ascorbic acid 2-phosphate (Sigma-Aldrich). Cell counts were performed at every passage and used to track population doublings over time in culture:

(1) $$\begin{array}{c} Population\;\;doublings=\frac{\log_{10}\left(cell\;\;count\;\;at\;\;passage\right)-\log_{10}\left(no.\;\;of\;\;cells\;\;initially\;\;seeded\right)}{\log_{10}\left(2\right)}. \end{array}$$

Cells of between 20 and 40 population doublings were used for all experiments. Four individual mDPSC clones that expanded sufficiently to allow multiple reproducible differentiation experiments were used in this study, each derived from a separate pulpal extraction (*n* = 4).

### 2.2. Neuronal Differentiation

mDPSC clones were seeded at 10,000 cells/cm^2^ on poly-d-lysine/laminin-coated culture surfaces in DMEM/F12 (1 : 1) containing L-glutamine and HEPES buffer, 100 units/mL penicillin, 100 *μ*g/mL streptomycin, and 1 × N2 supplement (all from Life Technologies), 1 × NEAA (Sigma-Aldrich), and 20 ng/mL basic fibroblast growth factor (bFGF) and 20 ng/mL epidermal growth factor (both from Peprotech). After 5 days, cultures were washed with PBS and changed to neurobasal medium supplemented with 100 units/mL penicillin, 100 *μ*g/mL streptomycin, and 2 mM L-glutamine (all from Life Technologies), 1 × NEAA (Sigma-Aldrich), and 10 ng/mL brain-derived neurotrophic factor, 10 ng/mL nerve growth factor, and 10 ng/mL neurotrophin-3 (all from Peprotech). RNA was extracted on days 0, 5, 10, and 15 of differentiation for use in qPCR and cells were fixed on day 15 for immunocytochemistry.

### 2.3. Oligodendrocyte Differentiation

Clonal mDPSC cultures were seeded at 10,000 cells/cm^2^ on poly-d-lysine-coated culture surfaces in DMEM containing 100 units/mL penicillin, 100 *μ*g/mL streptomycin, SATO supplement (16 *μ*g/mL putrescine, 62 ng/mL progesterone, 5 ng/mL sodium selenite, and 100 *μ*g/mL bovine serum albumin (BSA)), 50 *μ*g/mL holo-Transferrin, and 5 *μ*g/mL insulin (all from Sigma-Aldrich) and 10 ng/mL platelet-derived growth factor-aa and 20 ng/mL bFGF (both Peprotech). After 10 days differentiation cells were fixed for immunocytochemistry.

### 2.4. Isolation of mSTM Neurons

Mouse striatal (mSTM) neuronal tissues were dissected in PBS from P0 mice, digested using Accutase, and plated on poly-l-lysine-coated glass coverslips in Advanced DMEM/F-12 supplemented with 2 mM L-glutamine, 100 units/mL penicillin, 100 *μ*g/mL streptomycin, 1.8 mM CaCl_2_, 0.5 mM/L valproic acid, and 1 x B27-supplement (without vitamin A) (all from Life Technologies).

### 2.5. Reverse Transcriptase PCR

Total RNA was extracted using an RNeasy Mini Kit with on-column DNase digestion (QIAGEN) according to manufacturer's directions and cDNA synthesised using MMLV reverse transcriptase (Promega). PCR reactions were performed using GoTaq Polymerase (Promega) and product-specific primers (Supplemental Table 1 in Supplementary Material available online at http://dx.doi.org/10.1155/2016/1290561). RNA extracted from primary cultured E14.5 cortical neural stem cells, isolated as described previously [26], was used as a positive control.

### 2.6. Real Time Quantitative PCR

For qPCR readings, cDNA samples generated from three separate experiments per clone were used (*n* = 3) and each was measured in triplicate using an ABI Prism 7000 machine (Advanced Biosystems). Target-specific primers (Supplemental Table 2) were added to each cDNA sample together with Precision MasterMix with ROX and SYBRgreen (PrimerDesign). Dissociation curves were recorded to check for specificity of reactions and products were electrophoresed on 1.4% agarose gels in order to confirm product size. Relative changes in expression were calculated using the 2^−ΔΔCT^ method [27]. Statistical analyses were performed using Graphpad Prism Software.

### 2.7. Immunocytochemistry

Cells were fixed with 4% (w/v) paraformaldehyde for 15 min at room temperature and permeabilised in 0.1% (v/v) Triton X-100 for 10 min. Nonspecific antibody binding was blocked by incubating in 2% (w/v) BSA for 30 min. Cells were incubated overnight with the following primary antibodies: nestin (Santa Cruz), musashi (Life Technologies), microtubule-associated protein 2 (Map2 (Millipore)), neurofilament light chain NF-l (Abcam), Olig1 and Olig2 (both from Millipore), myelin basic protein (MBP (Abcam)), and *β*-actin (Cell Signalling). On the following day, complementary Alexa Fluor 488- and 594-conjugated secondary antibodies (Life Technologies) were applied. Glass coverslips were mounted using mounting media supplemented with DAPI stain (VectorLabs) and preparations imaged under a fluorescent microscope.

### 2.8. Patch Clamp Electrophysiological Recordings

Transmembrane currents of primary cultured mSTM neurons from days 3 to 21 of culture and mDPSCs neuronally differentiated for 15 days were recorded in conventional whole-cell configuration. The bath solution contained 135 mM NaCl, 5 mM KCl, 1.2 mM MgCl_2_, 1.25 mM CaCl_2_, 10 mM d-glucose, and 5 mM HEPES; pH was adjusted to 7.4 using NaOH. The pipette solution contained 117 mM KCl, 10 mM NaCl, 2 mM MgCl_2_, 1 mM CaCl_2_, 2 mM Na_2_ATP, 1 mM Na_2_GTP, 11 mM HEPES, and 11 mM ethylene glycol tetra acetic acid; free [Ca^2+^]_*i*_ was adjusted to 100 nM; pH was adjusted to 7.2 with KOH. All recordings were performed at room temperature (22 ± 0.5°C) using an Axopatch 200B amplifier and Digidata 1320 A/D interface (Axon Instruments). Holding voltages were set to −70 mV and transmembrane currents recorded using a voltage step protocol of 80 ms duration in voltage range from −120 to +80 mV. Series resistance and membrane capacitance were compensated ≈90%. Pipette resistances were ≈5–10 MΩ when filled with the pipette solution. All recordings were filtered with an 8-pole Bessel filter at 5 kHz and digitized at 10 kHz. Tetraethylammonium chloride (TEA) was purchased from Sigma.

### 2.9. Data Analysis

The patch clamp data were analyzed using Clampfit 9.0, Microsoft Office Excel 2003, and Microcal Origin 6.0 software. Transient inward Na^+^ currents were presented as peak values whereas outward steady-state K^+^ currents were presented as means. Current densities (pA/pF) were plotted against command voltage (mV). Statistical comparisons of the means were performed using independent *t*-test; differences were considered significant at *p* < 0.05.

## 3. Results

### 3.1. Isolation, Expansion, and Characterisation of Clonal mDPSC Cultures

**Figure 1:**
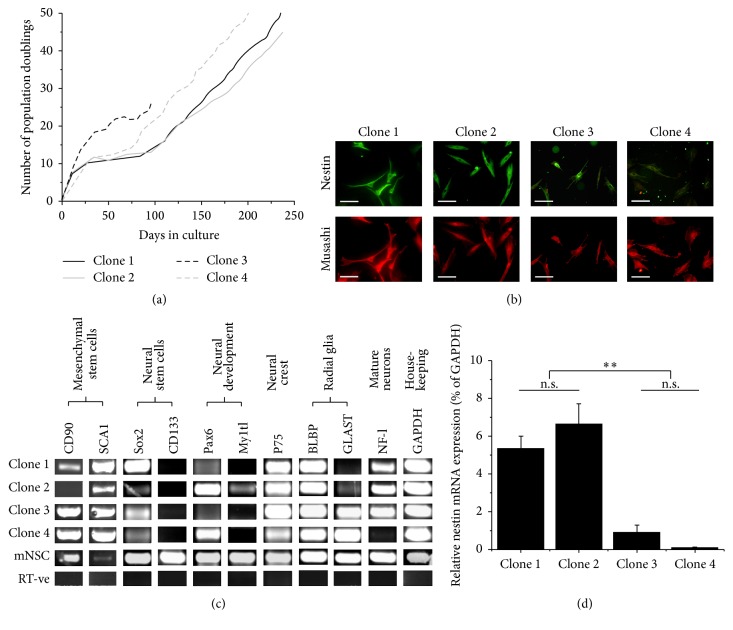
*In vitro* expansion and heterogeneity in the expression of developmental markers by clonal mDPSC cultures. (a) Single cell-derived clones, each expanded from a separate pulpal extraction, proliferated steadily for up to 240 days of culture reaching 50+ population doublings (*n* = 4 clones). Traces represent continuous culture growth from day of primary isolation, and cryopreserved cells continued to proliferate beyond the population doublings indicated. (b) Double immunostaining of clonal cultures for neural progenitor markers nestin and musashi. (c) RNA extracted from each clone between 20 and 40 population doublings was used in RT-PCR to identify clonal differences in the expression of RNA transcripts for CD90, stem cell antigen 1 (SCA1), glutamate aspartate transporter (GLAST), Sox2, Pax6, myelin transcription factor 1-like (Myt1l), P75, musashi, neurofilament light chain (NF-l), and CD133. (d) qPCR analysis of nestin mRNA expression by four mDPSC clones. Clones 1 and 2 were each individually found to express significantly higher levels of nestin than both clones 3 and 4. DPSC cultures were subsequently divided into strongly nestin-positive clones (clone 1 and clone 2) and weakly nestin-positive clones (clone 3 and clone 4). Nestin expression was calculated as a relative percentage of GAPDH ± SEM using the 2^−ΔΔCT^ method (*n* = 3, RNA samples extracted from three separate passages per clone between 16 and 40 population doublings). One-way ANOVA with Tukey-Kramer posttest: n.s. = not significant, ^*∗∗*^ *p* < 0.01. Scale bars = 100 *μ*m.

Dental pulp cells were successfully isolated and cultured from murine incisors. One day following isolation, sparsely distributed fibroblastic-like cells were identified growing on fibronectin-coated culture surfaces. A number of these rapidly expanded clonally to form discrete individual colonies. Clones were isolated at day 12 and found to expand over extended periods, some reaching 50+ population doublings, confirming a highly proliferative phenotype (Figure 1(a)). Presence of the neural progenitor markers nestin and musashi was identified within expanding clones using immunocytochemistry (Figure 1(b)). RNA was extracted from each clone between 20 and 40 population doublings and used in RT-PCR to identify further similarities in marker expression between four mDPSC clones and primary cultured murine neural stem cells (mNSCs) (Figure 1(c)). mDPSC clones were found to express a range of transcripts also expressed by NSCs including CD90, SCA1, GLAST, Sox2, Pax6, Myt1l, P75, BLBP, musashi, and NF-l. However, marked clonal differences were observed, with only the general stem cell marker SCA1, the neural crest-associated marker P75, and the radial glial protein BLBP being expressed by all mDPSC clones tested. No expression of CD133 was found in any mDPSC clone. Furthermore, clonal differences were identified in the mRNA expression levels of nestin. Based on a semiquantitative analysis, 8 out of 11 isolated clones appeared to express nestin mRNA transcripts at higher levels than the remaining 3 clones. However, only 4 of these clones continued to expand to allow the extraction of further RNA samples for a more accurate quantification of nestin expression and thus are subsequently described in this study. qPCR identified that clone 1 and clone 2 each expressed significantly higher levels (*p* < 0.01) of nestin transcripts than both clone 3 and clone 4 (Figure 1(d)). Subsequently, clones 1 and 2 were defined as high nestin-expressing clones and clones 3 and 4 as low nestin-expressing clones.

### 3.2. Neuronal-Like Differentiation

**Figure 2:**
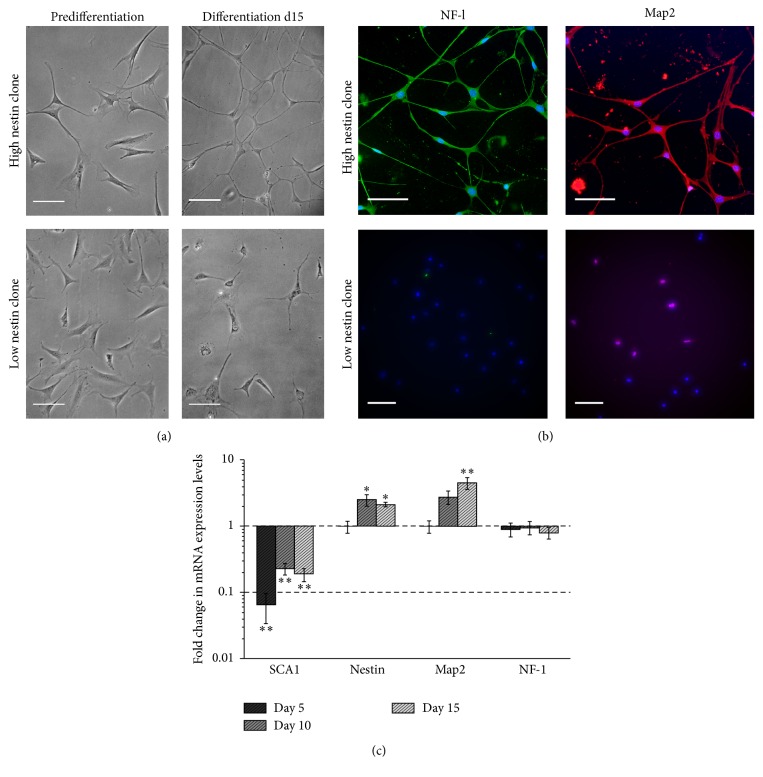
Only high nestin-expressing mDPSC clones possess the ability to differentiate into neuronal-like cells. (a) Representative phase contrast images of high (clone 2) and low (clone 3) nestin-expressing clones prior to and following 15 days of neuronal differentiation demonstrating a more neuronal-like morphology in high nestin-expressing clones with small refractive cell somas extending multiple interconnecting processes. (b) Immunocytochemical staining identified the presence of microtubule-associated protein 2 (Map2) and NF-l in high, but not low, nestin-expressing mDPSC clones following 15 days of neuronal differentiation. (c) Changes in mRNA expression of mesenchymal and neural markers during neuronal differentiation of high nestin-expressing mDPSC clones (clone 2). Expression levels of target genes were normalized against GAPDH and the 2^−ΔΔCT^ method for qPCR analysis used to calculate fold change in expression relative to predifferentiation cells on day 0 ± SEM (*n* = 3 independent differentiation experiments). One-way ANOVA with Dunnett multiple comparisons posttest to identify significant increases/decreases in expression compared to day 0 cells: ^*∗*^ *p* < 0.05 and ^*∗∗*^ *p* < 0.01. Scale bars = 100 *μ*m.

mDPSCs were typically bi/tripolar and fibroblastic-like in morphology prior to differentiation. Following 15 days of differentiation, clones initially identified as having high levels of nestin mRNA expression adopted a more neuronal-like phenotype with multiple neurite-like extensions. Conversely, no significant changes in morphology were observed in low nestin-expressing clones (Figure 2(a)). Immunocytochemical staining of the mature neuronal proteins Map2 and NF-l was identified in high, but not low, nestin-expressing mDPSC clones after 15 days of differentiation (Figure 2(b)). Changes in the mRNA expression of early and mature neuronal markers by high nestin-expressing clones over 15 days of differentiation were analyzed using qPCR (Figure 2(c)). By day 5 of differentiation, mRNA expression of SCA1 was strongly downregulated suggesting a transition from the default mesenchymal phenotype associated with mDPSCs. From day 5 onwards, after transferring to the neutrophin-containing maturation medium, expression of nestin and Map2 was seen to increase, indicating a more neuronal-like phenotype. mRNA expression levels for NF-l were found to be unchanged during the 15 days of differentiation.

**Figure 3:**
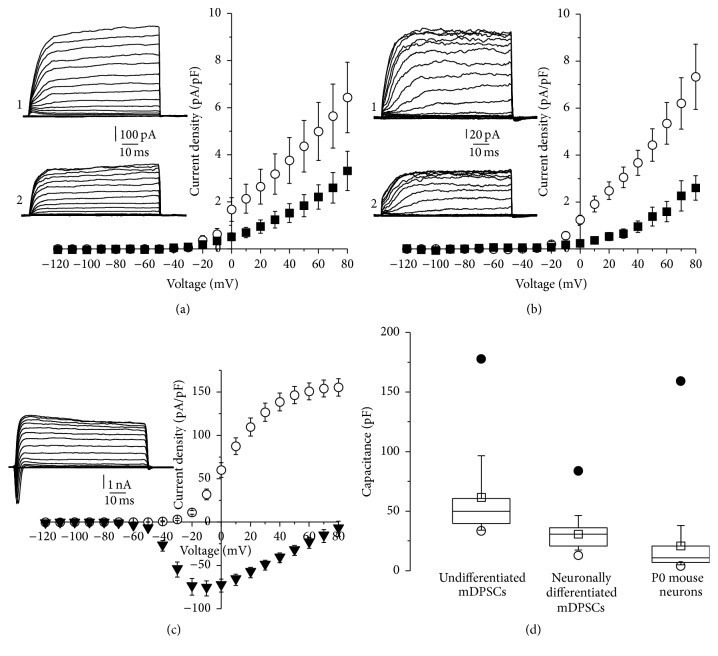
Neuronally differentiated high nestin-expressing mDPSCs show immature electrophysiology properties. Current density-voltage relationships of transmembrane K^+^ currents of undifferentiated (a) and neuronally differentiated (b) high nestin-expressing mDPSCs (clone 2) in the absence (○, inlet 1 illustrates exemplar trace of currents, *n* = 14  and  17 cells, resp.) and presence of TEA (1 mM) (■, inlet 2 illustrates exemplar trace of currents, *n* = 9 for each differentiation condition). (c) Current density-voltage relationships of transmembrane K^+^ current (○) and Na^+^ currents (▼) of mSTM neurons (*n* = 23 cells). Inlet illustrates exemplar trace of currents. The mean ± SEM current densities at +80 mV of undifferentiated (6.4 ± 1.5 pA/pF) and neuronally differentiated mDPSCs (7.3 ± 1.4 pA/pF) showed a significant difference in comparison with primary cultured mSTM neurons (155.4 ± 10.1 pA/pF): *p* < 3.0*E* − 13 and *p* < 6.0*E* − 15, respectively. (d) Comparison of capacitances of all three cell types. The mean values ± SEM (□) of undifferentiated mDPSCs (62.0 ± 10.2) and neuronally differentiated high nestin-expressing mDPSCs (30.7 ± 4.0), as well as undifferentiated mDPSCs and P0 mSTM neurons (20.9 ± 6.6), were considered as significantly different: *p* < 0.005 and *p* < 0.002, respectively. ● and ○ are maximal and minimal values, respectively.

Patch clamp recordings were taken to characterise the electrophysiological properties of high nestin-expressing mDPSC clones prior to, and after, 15 days of differentiation. Primary cultured mSTM neurons provided a positive control for comparison purposes. Only outward K^+^ currents were detected in mDPSCs, both before and after differentiation. These currents were effectively inhibited with 1 mM TEA, a nonselective blocker of K^+^ channels (Figures 3(a) and 3(b)). At the same time, both voltage-activated K^+^ and Na^+^ currents were recorded in P0 mSTM neurons (Figure 3(c)). At a voltage of +80 mV, significantly higher K^+^ current densities were recorded in P0 mSTM neurons (155.4 ± 10.1 pA/pF) compared to undifferentiated (6.4 ± 1.5 pA/pF) and neuronally differentiated mDPSCs (7.3 ± 1.4 pA/pF): *p* < 3.0*E* − 13 and *p* < 6.0*E* − 15, respectively. No significant difference was found between the current densities of undifferentiated and neuronally differentiated mDPSCs; however, membrane capacitance varied dramatically (Figure 3(d)). Differentiated mDPSCs (30.7  ±  4.0) possessed statistically significant lower membrane capacitances than undifferentiated mDPSCs (62.0 ± 10.2): *p* < 0.005. This lower capacitance was directly comparable with p0 mSTM neurons (20.9 ± 6.6), with no significant difference observed (*p* > 0.2). Together, these results suggest that, despite appropriate morphology, the presence of mature neuronal proteins after 15 days of differentiation, and a comparable cell capacitance to primary cultured neurons, high nestin-expressing mDPSC clones maintain an electrophysiologically immature phenotype.

### 3.3. Oligodendrocyte-Like Differentiation

**Figure 4:**
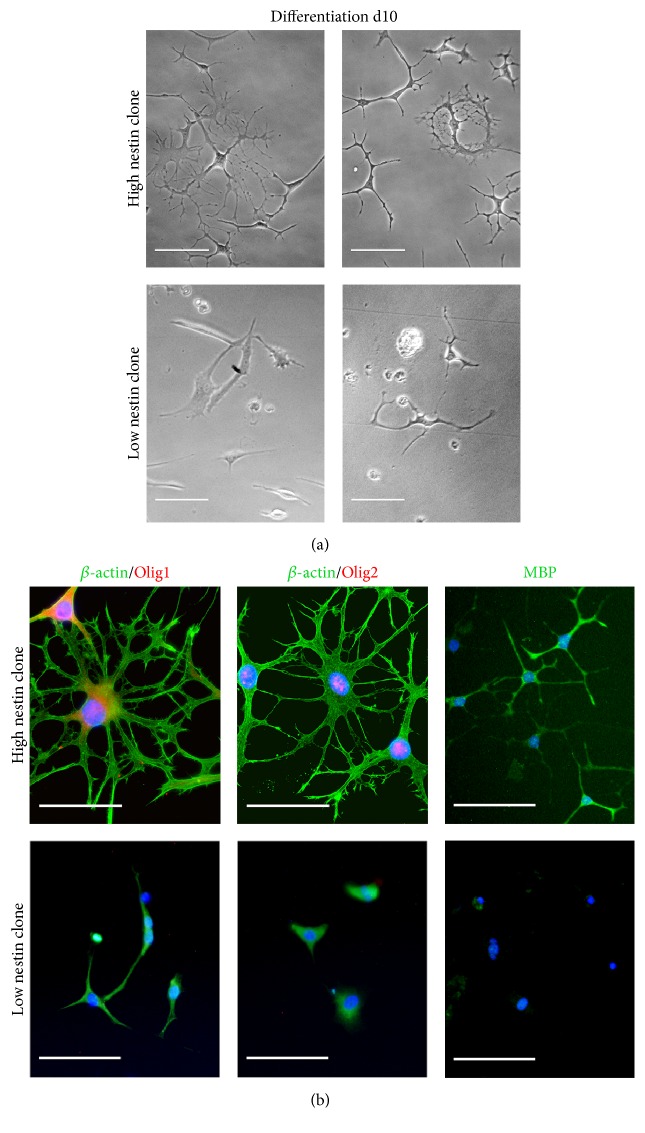
Only high nestin-expressing mDPSC clones display the ability to differentiate into oligodendrocyte-like cells. (a) Representative phase contrast images of high (clone 2) and low (clone 3) nestin-expressing clones following 15 days of oligodendrocyte-like differentiation. Clones with higher levels of nestin mRNA were found to adopt a more highly branched oligodendrocyte-like morphology compared to lower nestin-expressing clones. (b) Immunocytochemical staining identified the presence of myelin basic protein (MBP) and the oligodendrocyte transcription factors Olig1 and Olig2 in high, but not low, nestin-expressing mDPSC clones following 10 days of differentiation. *β*-actin staining was performed to demonstrate highly branched morphology. Scale bars = 100 *μ*m.

Following 10 days of differentiation, clones with initially high levels of nestin were seen to adopt a highly branched oligodendrocyte-like morphology. Although some branching was observed, low nestin-expressing clones largely failed to survive 10 days of differentiation (Figure 4(a)). Immunocytochemical staining of myelin basic protein (MBP), Olig1, and Olig2 was only observed in high nestin-expressing mDPSC clones (Figure 4(b)). The expression of oligodendrocyte-associated proteins, together with appropriate morphology, suggests that this novel protocol may be used to derive an oligodendrocyte-like phenotype from mDPSCs with high levels of nestin expression.

## 4. Discussion

In this study we have identified heterogeneity in the ability of single cell-derived clonal cultures of mDPSCs to differentiate into neuronal-like and glial-like cells. Those clones possessing the highest levels of mRNA expression for the neuronal progenitor-associated intermediate filament protein, nestin, showed a greater potential for differentiation down both neural lineages. Although some evidence of variability in the neural differentiation potential of heterogeneous DPSC has been previously described [18, 22], our findings suggest that nestin may act as a suitable marker for which to screen DPSC cultures* in vitro* prior to use in neural tissue engineering applications. The problems associated with cellular heterogeneity are increasingly becoming recognised in the stem cell research field and gaining a fuller understanding of the variability within transplantable populations will help maximise the potential of any stem cell-based therapy.

Prior to differentiation, the expression of a range of developmental and neural progenitor markers by single cell-derived mDPSC cultures was extensively analyzed and compared with primary mNSCs. Although mDPSCs were found to express a number of markers also associated with NSCs including Sox2, Pax6, GLAST, BLBP, nestin, and NF-l, expression patterns were highly variable between clones, demonstrating the degree of heterogeneity that exists within the mixed populations of DPSCs typically used for neural transplantation studies [10–15]. Importantly, only those clones identified with high levels of nestin mRNA expression displayed the ability to differentiate into a neuronal-like phenotype based on cell morphology and increased expression levels of the more mature neuronal marker Map2. To test the electrophysiological properties of these cells, patch clamp recordings were made. The electrical properties of neuronal-like cells derived from murine DPSCs remain largely uncharacterised in contrast to human DPSCs in which voltage-activated Na^+^ and K^+^ currents and ATP-activated Ca^2+^ surges have been recorded [17, 18, 20, 21]. In the only previous functional study using rodent DPSCs, mixed populations of mDPSCs differentiated using an established protocol displayed voltage-activated Ca^2+^ but not K^+^ or Na^+^ currents, directly contradicting recordings taken when the same protocol was applied to human hDPSCs [18, 19]. Single cell-derived cultures of high nestin-expressing mDPSCs differentiated using the protocol described in this report, on the other hand, display TEA-sensitive voltage-gated K^+^ currents, demonstrating the presence of functional voltage-activated K^+^ channels in neuronally differentiated rodent DPSCs for the first time. Although the amplitude of these currents is reduced when compared to mSTM neurons, similar membrane capacitances were measured for each cell type. This reduction in capacitance is indicative of cells with an ability to store electrical charge directly comparable to primary cultured striatal neurons, confirming a more neuronal-like phenotype after differentiation. Although a fully functional phenotype with the ability to fire action potentials has yet to be derived from either human or rodent DPSCs, there is sufficient evidence here to suggest that high nestin-expressing mDPSCs may be promoted to differentiate, at least partially, along this lineage. However, further steps will be required to obtain a more mature neuronal-like phenotype and future studies might focus on incorporating a supporting cell type in coculture to provide appropriate trophic factor support for the development and maturation of functional properties, for example, astrocytes [28, 29].

Oligodendrocyte-like differentiation of DPSCs has only previously been described* in vivo*, following mixed population transplantation into a rat model of spinal cord injury [12]. Using a novel protocol adapted from those used in the culture and differentiation of oligodendrocyte progenitor cells (OPCs) [30, 31], mDPSC clones with high levels of nestin mRNA expression adopted a highly branched oligodendrocyte-like morphology and stained positive for oligodendrocyte markers Olig1, Olig2, and MBP. Despite the expression of MBP in differentiated mDPSCs, there was no observation of membranous sheets associated with mature myelinating oligodendrocytes* in vitro* [30, 32]. This suggests that, similar to neuronal differentiation, high nestin-expressing mDPSCs are able to differentiate partially to an immature premyelinating phenotype, but further differentiation steps may be required for full functionality. Nevertheless, the development of this protocol represents a significant finding and may provide a useful* in vitro* research tool for further studies into mechanisms through which DPSCs may promote central nervous system repair and regeneration.

Unlike bone marrow, another common source of mesenchymal stem cells, dental pulp is a nonhaematopoetic tissue and clonal DPSC cultures may be more lineage-restricted in nature [9]. Their highly heterogeneous nature is purported to be attributable to multiple populations of progenitor cells residing in different locations of the pulp which may possess different proliferative and differentiation capabilities. Different niches have been identified* in situ* associated with the vasculature, within the pulpal stroma, in the subodontoblast layer and amongst peripheral nerve-associated glial cells [33–38]. During development, the dental pulp and central nervous system both derive from the embryonic ectoderm. Following neurulation, multipotent neural crest cells migrate away from the neural tube into developing craniofacial tissues. At the initiation of tooth morphogenesis, these cells populate the underlying mesenchyme, eventually giving rise to the cellular components of pulpal tissue [39]. Multipotent adult DPSCs that maintain neural crest stem cell characteristics and may represent a source of cells with greater potential for neuronal and glial differentiation given their developmental origin have been isolated from different niches within the pulp [25, 38, 40, 41]. A recent study compared the proliferative and differentiation potentials of human DPSCs based on the expression of the pericyte-associated cell surface antigen CD34 [42]. Only CD34^+^ hDPSCs were found to express nestin and possess the ability to differentiate down neuronal lineages, similar to the high nestin-expressing mDPSC described here, suggesting that they may be neural crest in origin and derived from a perivascular-associated niche. It may prove beneficial to select for such DPSCs in future neural tissue engineering studies.

Together, the results presented herein suggest that mRNA levels of nestin may be indicative of the potential of mDPSCs for neuronal-like and oligodendrocyte-like differentiation. Nestin expression is associated with stem cells in the developing neural tube as well as specific subtypes of OPCs [43, 44]. As such, its link to mDPSCs with neuronal and oligodendrocyte-like differentiation capabilities fits. However, nestin-positive cells make up only a small fraction of the total cellular component of dental pulp, less than 3.5% reported in isolates from rat incisors [45]. Most published studies utilise such mixed populations of cells and so likely contain a significant proportion of other cell types, perhaps explaining previous inconsistencies in response to neuronal differentiation cues [18, 22]. The use of clonally derived cultures allows investigations to be carried out at the single cell level and the subsequent identification of differences between individual clonal cell lines. Large differences in the proliferation and mineralisation potential of clonal DPSC cultures have been previously reported in this manner [1, 9]. Similarly, differences in the neuronal-like and oligodendrocyte-like differentiation potential of mDPSC clones are reported here. The use of single cell-derived clones is unlikely to be therapeutically applicable due to scalability issues within short time frames. However, clonal cultures serve as an extremely useful research tool to identify desirable properties of cells within mixed populations. In future studies, the screening of single cell-derived clones on a larger scale to that described in this report will serve to further our understanding of cellular heterogeneity and its implications for the development of stem cell-based therapies.

## 5. Conclusions

Significant heterogeneity exists between clonal cultures of mDPSCs and clones with comparatively higher levels of nestin expression possess a greater capacity for differentiation into neural lineages. These findings help explain previous reports of only small numbers of transplanted DPSCs adopting neuronal-like and glial-like phenotypes after transplantation, as well as inconsistencies in* in vitro* differentiation studies. In conclusion, high nestin-expressing DPSCs may represent a more desirable cell source for promoting central nervous system repair and regeneration.

## Supplementary Material

This supplementary material includes a list of all primers used in this body of work and the accession numbers of the genes which they target. Sequences, expected PCR product sizes and optimised annealing temperatures are also detailed for each primer pair.
