# Supplementary material for: Clonal Heterogeneity in the Neuronal and Glial Differentiation of Dental Pulp Stem/Progenitor Cells
Source: Stem Cells Int. 2016 May 26;2016:1290561. doi: 10.1155/2016/1290561 (PMC4899607; doi:10.1155/2016/1290561)
Supplement: Supplementary file 1 — This supplementary material includes a list of all primers used in this body of work and the accession numbers of the genes which they target. Sequences, expected PCR product sizes and optimised annealing temperatures are also detailed for each primer pair. [file 1290561.f1.pdf]

Supplemental tables

Supplemental table 1 – Primers used for rt-PCR

Product	Accession no.	Primer Sequences (5' → 3)	Product Size (bp)	Tm (°C)
BLBP	NM_021272.3	f: GGACACAATGCACATTCAAGAA<br>r: CCGAACCACAGACTTACAGTTT	101	60
CD90	NM_009382.3	f: GCCTGACAGCCTGCCTGGTGAACCAAA<br>r: TGCCGCCACACTTGACCAGCTTGTCTCT	330	60
CD133	NM_008935.2	f: GAATGCGCCATGCAGGAGGAAGTGCTT<br>r: GGCTGCTCCCCAGACTGCTTAGGCTTG	542	60
GAPDH	NM_008084.2	f: AGACGGCCGCATCTTCTTGTGCAGTGC<br>r: ACATACTCAGCACCGGCCTCACCCCA	326	60
GLAST	NM_148938.3	f: ACCAAAAGCAACGGAGAAGAG<br>r: GGCATTCCGAAACAGGTAACTC	144	60
Musashi	NM_008629.1	f: GGGGTGGATAAAGTGCTGGCGCAATCG<br>r: CGCTCTACACGGAATTCGGGGAACTGGT	533	60
Myt1l	NM_001093775.1	f: TGGTCACGTCAGTGGCAAATA<br>r: TGCAAATGGTTTTCGCTTGGG	121	60
NF-I	NM_010910.1	f: CAAGAGCCGCTTCACCGTGCTAACCGA<br>r: CGCTGGTTATGCTACCCACGCTGGTGA	392	60
P75	NM_033217.3	f: CTCAGATGAAGCCAACCACG<br>r: CCTTGTGATCCATCGGCCA	133	60
Pax6	NM_001244198.1	f: TACCAGTGTCTACCAGCCAAT<br>r: TGCACGAGTATGAGGAGGTCT	194	60
SCA1	NM_010738.2	f: ACCTGCCCCTACCCTGATGGAGTCTGT<br>r: TGTGTGCCTCCAGGGTCATGAGCAGCA	522	60
SOX2	NM_011443.3	f: GCGGAGTGGAAACTTTTGTCC<br>r: CGGGAAGCGTGTACTTATCCTT	157	60

Supplemental table 2 – Primers used for qPCR

Product	Accession no.	Primer Sequences (5' → 3)	Product Size (bp)	Tm (°C)
GAPDH	NM_008084.2	f: AGGTCGGTGTGAACGGATTTG<br>r: TGTAGACCATGTAGTTGAGGTCA	123	60
Map2	NM_001039934.1	f: TGACACTTGGGACCTGGACGAGTAT<br>r: ACACCACTTCTTCAACCAACGCTCA	105	60
Nestin	NM_016701.3	f: CCAAAGAGGTGTCCGATCATC<br>r: CTCCCTTCTTCTTCATCAGCATCT	147	60
NF-l	NM_010910.1	f: TGAGAAGCACGAAGAGCGAGATGG<br>r: TGAAACTGAGCCTGGTCTCTTCGC	135	60
SCA1	NM_001271416.1	f: GAGGCAGCAGTTATTGTGGAT<br>r: ACCCAGGATCTCCATACTTTCA	99	60
